# hnRNPK inhibits GSK3β Ser9 phosphorylation, thereby stabilizing c-FLIP and contributes to TRAIL resistance in H1299 lung adenocarcinoma cells

**DOI:** 10.1038/srep22999

**Published:** 2016-03-14

**Authors:** Xuejuan Gao, Junxia Feng, Yujiao He, Fengmei Xu, Xiaoqin Fan, Wensi Huang, Haiting Xiong, Qiuyu Liu, Wanting Liu, Xiaohui Liu, Xuesong Sun, Qing-Yu He, Qihao Zhang, Langxia Liu

**Affiliations:** 1 Key Laboratory of Functional Protein Research of Guangdong Higher Education Institutes, Institute of Life and Health Engineering, Jinan University, Guangzhou 510632, China; 2 Department of Nephrology, Huadu District People’s Hospital, Southern Medical University, 22 Baohua Road, Guangzhou 510800, China; 3 Longgang Central Hospital, ENT Hospital, Shenzhen ENT Institute, Shenzhen 518116, China; 4 Department of Pathology, Henan Provincial People’s Hospital, People’s Hospital of Zhengzhou University, 7 Weiwu Road, Zhengzhou 450003, China; 5 Institute of Biomedicine, and National Engineering Research Center of Genetic Medicine, Jinan University, Guangzhou 510632, China; 6 Guangdong Provincial Key Laboratory of Bioengineering Medicine, Jinan University, Guangzhou 510632, China

## Abstract

c-FLIP (cellular FLICE-inhibitory protein) is the pivotal regulator of TRAIL resistance in cancer cells, It is a short-lived protein degraded through the ubiquitin/proteasome pathway. The discovery of factors and mechanisms regulating its protein stability is important for the comprehension of TRAIL resistance by tumor cells. In this study, we show that, when H1299 lung adenocarcinoma cells are treated with TRAIL, hnRNPK is translocated from nucleus to cytoplasm where it interacts and co-localizes with GSK3β. We find that hnRNPK is able to inhibit the Ser9 phosphorylation of GSK3β by PKC. This has the effect of activating GSK3β and thereby stabilizing c-FLIP protein which contributes to the resistance to TRAIL in H1299 cells. Our immunohistochemical analysis using tissue microarray provides the clinical evidence of this finding by establishing a negative correlation between the level of hnRNPK expression and the Ser9 phosphorylation of GSK3β in both lung adenocarcinoma tissues and normal tissues. Moreover, in all cancer tissues examined, hnRNPK was found in the cytoplasm whereas it is exclusively nuclear in the normal tissues. Our study sheds new insights on the molecular mechanisms governing the resistance to TRAIL in tumor cells, and provides new clues for the combinatorial chemotherapeutic interventions with TRAIL.

Lung cancer is the leading cause of cancer-related death in the world. Among all cases, more than 85% of them are non-small cell lung cancers (NSCLC)^1^. NSCLC patients are usually inappropriate for surgical intervention and therefore require systemic chemotherapy and radiation therapy. However, very poor prognosis has been observed for the lung cancer patients due to the chemotherapy resistance. Development of effective therapeutic strategies aiming to overcome the drug resistance is therefore required to improve the prognosis and survival of lung cancer patients^2^. During the past years, dealing with the chemotherapy resistance to the tumor necrosis factor (TNF)-related apoptosis-inducing ligand (TRAIL) has become a subject of interest for the worldwide researchers^3^,^4^,^5^,^6^. TRAIL is a promising therapeutic agent that selectively causes apoptosis in cancer cells while without toxicity toward normal human cells tested^7^,^8^. Soluble TRAIL as well as agonistic antibodies against TRAIL-receptor are currently in clinical trials^9^. Meanwhile, approximately 50% of human cancer cell lines and most of human primary tumor cells have been reported to be resistant to TRAIL, which is the cause of the very limited therapeutic efficacy of the latter^10^. Hence, elucidating the molecular mechanisms of the resistance to TRAIL will help to find out the effective strategies for sensitizing cancer cells to TRAIL-induced apoptosis^11^.

TRAIL is a member of the tumor necrosis factor (TNF) family, which induces apoptosis through binding to its death receptor TRAIL-R1 (DR4) and TRAIL-R2 (DR5), and activating the death receptor signaling pathways^12^,^13^. After binding to TRAIL, its receptors oligomerize and recruit the cytoplasmic proteins FADD (Fas-associated death domain protein) and procaspase-8 (or procaspase-10) to form the death-inducing signaling complex (DISC)^9^,^14^. The auto-activation of the caspase 8 in the complex results in the subsequent activation of effector caspases, including caspases 3, 6, and 7, and finally leads to cell apoptosis^9^,^15^.

TRAIL-induced death receptor pathway is regulated by various factors. Among these factors, cellular FLICE-inhibitory protein (c-FLIP) is considered to be a master anti-apoptotic regulator and resistance factor^16^,^17^,^18^. c-FLIP shares structural homology with procaspase-8 but does not contain the catalytic site as the latter. It can be therefore recruited to DISC through association with FADD to competitively inhibit the caspase 8 activation and acts as key suppressor of the death receptor signaling pathway^16^,^19^. The increased expression of c-FLIP is detected in a wide range of cancers^20^,^21^, and positively correlates with the resistance of cancer cells to death receptor ligands^22^. Conversely, the decreased expression of c-FLIP by chemicals or siRNA sensitizes cancer cells to death receptor-induced apoptosis^16^,^22^,^23^. Both c-FLIP_L_ (55 kD) and p43 c-FLIP (43 kD, the caspase-8 processed N-terminal fragment of c-FLIP_L_) could function as an apoptosis suppressor, with more efficiency of the latter^24^,^25^,^26^,^27^.

The ubiquitous serine/threonine kinase Glycogen synthase kinase beta (GSK3β) is another key regulator of apoptosis. GSK3β is thought to facilitate the mitochondrial intrinsic apoptotic pathway while block death receptor-induced apoptosis^28^. Inhibition or deletion of GSK3β has been reported to sensitize death receptor-induced apoptosis in numerous tumor cells^29^,^30^,^31^,^32^. Notably, inhibition of GSK3 by Celecoxib promoted the degradation of c-FLIP and death receptor-induced apoptosis, suggesting that GSK3 might stabilize c-FLIP and antagonized tumor resistance to TRAIL^33^.

We have previously identified hnRNPK (heterogeneous nuclear ribonucleoprotein K) as a putative interacting partner of GSK3β^34^. hnRNPK is a well conserved DNA and RNA binding protein and shares with several other RNPs the triple K-homology domain. hnRNPK shuttles between nucleus and cytoplasm and regulates gene expression at multiple levels^35^,^36^. The expression of hnRNPK is aberrantly increased in a variety of cancers^37^,^38^,^39^,^40^, and it has been reported that hnRNPK negatively regulated the TRAIL-induced apoptosis through up-regulating the transcriptional level of c-FLIP^41^.

The physical interaction between two antagonists of TRAIL-induced apoptosis: hnRNPK and GSK3β, together with their respective functional interactions with c-FLIP have prompted us to investigate the functional relationship among these three proteins in the resistance of TRAIL-induced apoptosis. In this study, we focused on the possible role of hnRNPK-GSK3β interaction in the regulation of the protein stability of c-FLIP, as well as the related functional consequence on the resistance to TRAIL-induced apoptosis of lung cancer cells.

## Results

### Interaction and co-localization of GSK3β with hnRNPK in H1299 lung adenocarcinoma cells

**Figure 1:**
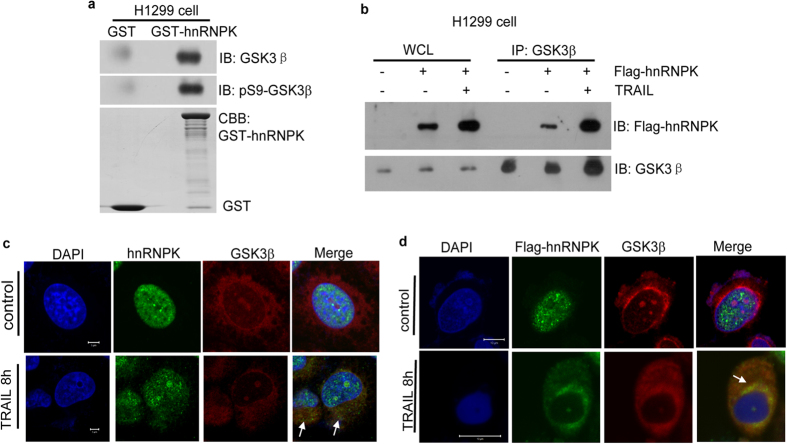
Interaction and co-localization of GSK3β with hnRNPK in H1299 cells. (**a**) GST-hnRNPK pull-down assay. H1299 cell lysates were incubated with GST or GST-hnRNPK fusion proteins immobilized on glutathione sepharose beads as described. Western blotting was then performed with GSK3β and phospho-GSK3β (Ser9) antibodies to detect the presence of GSK3β and phospho-GSK3β (Ser9) in the bound fractions. The amounts of recombinant proteins GST and GST-hnRNPK were estimated by CBB staining. (**b**) Co-immunoprecipitation assay of the interaction of GSK3β with hnRNPK. H1299 cells were transfected with Flag-hnRNPK or empty Flag-vector as indicated and treated with TRAIL (20 ng/ml, 8 hours). The existence of Flag-hnRNPK and GSK3β in the Sepharose-beads bound fractions were analyzed by immunoblot (IB) with Flag and GSK3β antibodies. WCL: whole cell lysates. (**c**) Subcellular localization of GSK3β and hnRNPK in H1299 cells after TRAIL treatment. H1299 cells treated with or without TRAIL (20 ng/ml, 8 hours) were fixed and stained with both GSK3β and hnRNPK antibodies. The Alexa Fluor 488 and Alexa Fluor 594 conjugated secondary antibody were used. Arrows: the co-localization of the two proteins. Bar: 5 μm. (**d**) Subcellular localization of GSK3β and ectopically expressed hnRNPK in H1299 cells after TRAIL treatment. H1299 cells transfected with Flag-hnRNPK were treated with TRAIL (20 ng/ml, 8 hours), then fixed and stained with both GSK3β and Flag antibodies. The same secondary antibodies used in (**c**) were used. Arrows: the co-localization of the two proteins. Bar: 10 μm.

We have previously demonstrated that GSK3β interacts with hnRNPK in HepG2 hepatocellular carcinoma cells^34^. Here, we firstly used GST pull-down assays to demonstrate this interaction using the cellular lysate of lung adenocarcinoma cancer H1299 cells. As shown in Fig. 1a, purified GST-hnRNPK was capable to specifically pull down the endogenous GSK3β in H1299 cell lysate. Interestingly, both anti-GSK3β antibody and anti-p9-GSK3β antibody were able to reveal the GSK3β trapped by the purified GST-hnRNPK. This raised the uncertainty about the phosphorylation state of the trapped GSK3β. Indeed, the GSK3β molecules interacting with GST-hnRNPK could be both Ser9 phosphorylated and unphosphorylated, or they could be only Ser9 phosphorylated and recognized by both the anti-GSK3β and anti-p9-GSK3β antibodies. These two different situations might respectively reflect the two possibilities where the Ser9 phosphorylated and unphosphorylated GSK3β interact equally or differentially with hnRNPK. To clarify this point, we have performed GST-pull down assays using H1299 cell lysates incubated respectively with either GST-GSK3β *wt* or GST-GSK3β S9A, a Ser9-phospho-defective mutant, and compared the quantity of hnRNPK trapped by these two forms of GSK3β. Our results shown in Fig. S1 indicate that GST-GSK3β *wt* and GST-GSK3β S9A displayed similar affinities vis-à-vis hnRNPK in H1299 cell lysates. Therefore, Ser9 phosphorylation *per se* might not be a factor directly affecting the affinity between GST-GSK3β and hnRNPK. The interaction between GSK3β and hnRNPK was then confirmed by co-immunoprecipitation experiments using the H1299 cells transfected by Flag-hnRNPK. As shown in Fig. 1b, Flag-hnRNPK co-immunoprecipited with GSK3β by anti-GSK3β antibody in the cell lysate of H1299 cells either treated or not by TRAIL. Confocal immunofluorescence assays were then performed to confirm this result by showing the co-localization of GSK3β and hnRNPK in the cytoplasm of H1299 cells. Figure 1c,d demonstrate that both endogenous and ectopically expressed hnRNPK could be partially translocated from nucleus to cytoplasm upon TRAIL treatment, and co-localized with GSK3β that was mainly cytoplasmic.

### Both GSK3β and hnRNPK antagonized TRAIL-induced apoptosis

**Figure 2:**
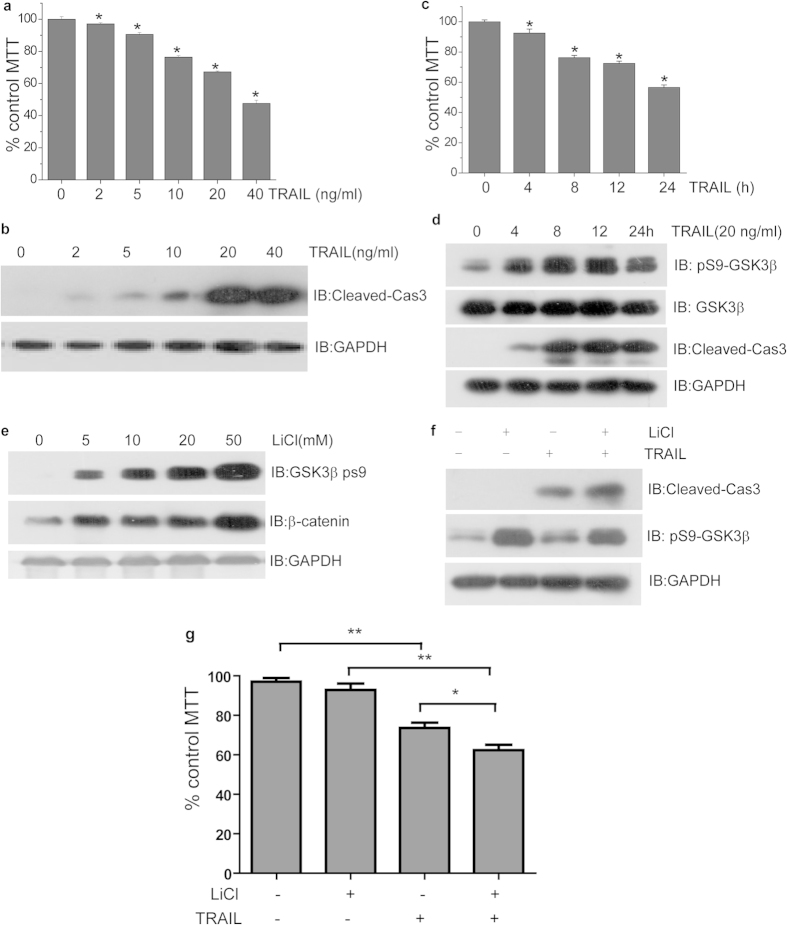
Both GSK3β and hnRNPK antagonized TRAIL-induced apoptosis. (**a**) MTT assays of cellular viability with TRAIL treatment at different concentrations. H1299 cells were seed into 96-well plate (4000 cells per well) and treated with TRAIL at increasing concentrations as indicated for 24 hours. MTT assays were then performed as described in Method section. The cellular viability was monitored by the absorbance of formazan crystals at 570 nm. The absorbance of the control group was set as 100%. *p < 0.05 (compared with control group). (**b**) Cleaved-Cas3 level in H1299 cells treated with TRAIL at various concentrations. H1299 cells were treated with TRAIL within the range of 2–20 ng/ml for 24 hours and harvested for Western blot analysis using cleaved-Cas3 antibody. (**c**) MTT assays of cellular viability after TRAIL treatment at different timescales as indicated. H1299 cells were treated with TRAIL at concentrations of 20 ng/ml for 4–24 hours, then analyzed with MTT using the same methods with (**a**). *p < 0.05 (compared with control group). (**d**) Cleaved-Cas3 level in H1299 cells treated with TRAIL during different timescales. H1299 cells were treated with TRAIL at concentrations of 20 ng/ml for 4–24 hours and analyzed by Western blotting using cleaved-Cas3 antibody. (**e**) The effect of LiCl on the activity of GSK3β in H11299 cells. Cells were treated with LiCl at various concentrations as indicated for 8 hours and harvested for Western blotting analysis with the indicated antibodies. (**f,g**) LiCl enhanced the apoptotic effect of TRAIL. H1299 cells treated with either LiCl (20 mM, 8 hours) or TRAIL (20 ng/ml, 8 hours) alone, or the combination of LiCl and TRAIL, were analyzed by Western blotting with the indicated antibodies (**f**), or by MTT assays (**g**). *p < 0.05. **p < 0.01.

In order to investigate the functional importance of GSK3-hnRNPK interaction in the TRAIL-induced apoptosis of H1299 cells, we searched at first to determine the optimal concentration and timing of TRAIL treatment on H1299 cells. A TRAIL dose-response experiment has been performed. H1299 cells were treated during 24 hours with increasing doses of TRAIL, and then analyzed by MTT assays for cellular viability and by Western blotting for the level of cleaved Caspase 3, both indicators of the apoptotic degree of cells. Our results in Fig. 2a,b showed that, when used within the range of 2–20 ng/ml, TRAIL induced apoptosis of H1299 cells in a dose-dependent manner, with a marked effect observed at 20 ng/ml, as judged by both the cleaved caspase 3 level and the cell death rate. This concentration was then used for another experiment destined to determine the timing of the treatment. Cells were harvested at different time points after TRAIL stimulation and analyzed as above for the apoptotic state of the cells. Our experiment suggested that 8–12 hours of TRAIL treatment were sufficient to induce significant apoptosis in H1299 cells (Fig. 2c,d). Subsequently, we attempted to assess the role of GSK3β in the TRAIL-induced apoptosis of H12199 cells by using LiCl that is a specific inhibitor of GSK3β kinase activity and inducer of the Ser9 phosphorylation of GSK3β^42^. In our experiment, increasing doses of LiCl, ranging from 5 mM to 50 mM, indeed resulted in the increasing level of GSK3β Ser9 phosphorylation, which was also confirmed by the increasing level of β-catenin whose degradation is an indicator of the kinase activity of GSK3β (Fig. 2e). The concentration of 20 mM was then chosen for the subsequent experiments of LiCl treatment. As described in Materials and Methods, we have treated H1299 cells separately or jointly with 20 mM LiCl and 20 ng/mL TRAIL, then examined the level of total GSK3β, Ser9 phosphorylated GSK3β, and cleaved Caspase 3 in these cells by Western blotting using specific antibodies (Fig. 2f). Our experiments showed that LiCl was capable to enhance the effect of TRAIL to induce apoptosis, reflected by the increased level of cleaved Caspase 3. MTT assays were then performed with This preliminary result conforming the pro-survival role of active GSK3β under the treatment of TRAIL was consistent with other reports^31^,^43^.

**Figure 3:**
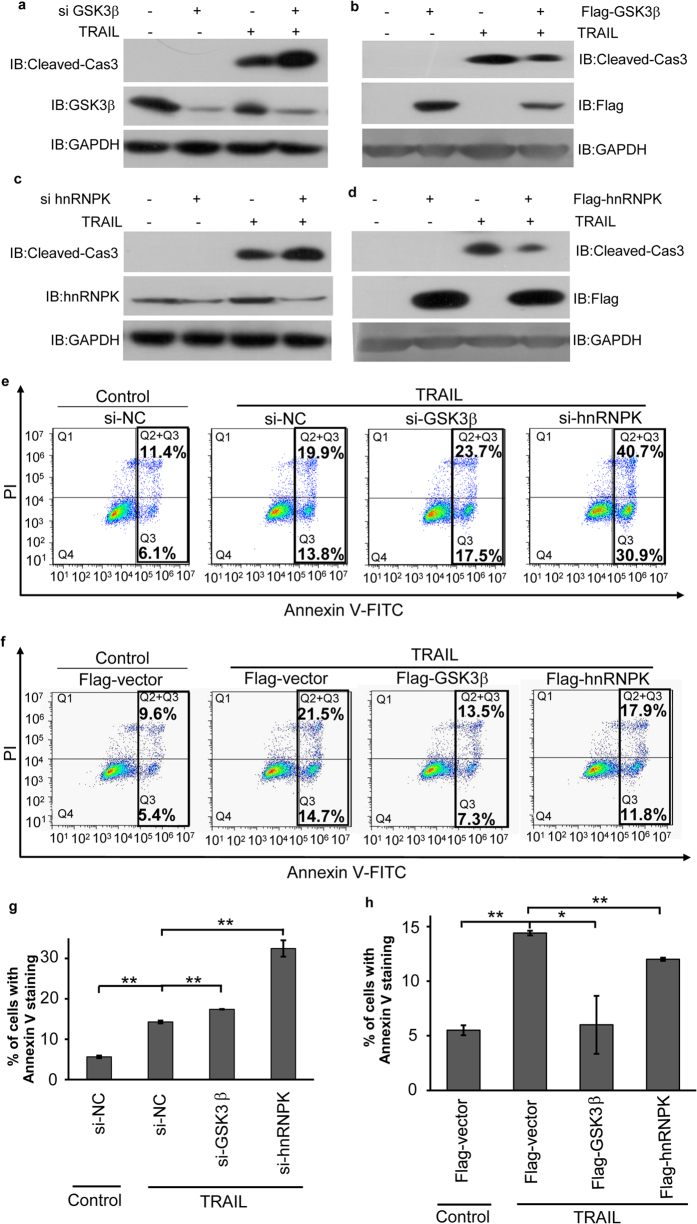
Both GSK3β and hnRNPK antagonized TRAIL-induced apoptosis. H1299 cells were transfected with siRNA against GSK3β (**a**), Flag-GSK3β (**b**), siRNA against hnRNPK (**c**), or Flag-hnRNPK (**d**), and treated with TRAIL (20 ng/ml, 8 hours), then subjected to Western blot analysis with the indicated antibodies. (**e,f**) Analysis of the cell death rate by Flow cytometry. H1299 cells transfected with GSK3β siRNA or hnRNPK siRNA (**e**), or transfected with Flag-GSK3β or Flag-hnRNPK (**f**) were treated with TRAIL (20 ng/ml, 8 hours) and analyzed using Annexin V/PI double staining with a FACS Calibure flow cytometer. (**g,h**) Statistical analyses of the experiments shown in (**e**,**f**) respectively. The analyses were performed with the results of three independent replicates for each experiment. *p < 0.05.

To further investigate the functions of both GSK3β and hnRNPK in TRAIL-induced tumor cell apoptosis, we have either knocked down or overexpressed GSK3β or hnRNPK respectively by transient transfection of H1299 cells with corresponding siRNA or Flag-tagged expression plasmids. Cells were then examined by Western blotting using specific antibodies against Flag tag, GSK3β, hnRNPK and cleaved Caspase 3, or by FACS with Annexin V/PI double staining (Fig. 3a–h). As shown, knockdown of either GSK3β or hnRNPK significantly increased the level of the cleaved Caspase 3 in Western blotting analyses and also the cell apoptotic rates in FACS assays, whereas the opposing effects were observed when cells were transfected by Flag-GSK3β or Flag-hnRNPK (Fig. 3a–h). These results indicated that both GSK3β and hnRNPK antagonized TRAIL-induced apoptosis in H1299 cells.

### hnRNPK down-regulates the Ser9 phosphorylation of GSK3β by PKC in H1299 cells

**Figure 4:**
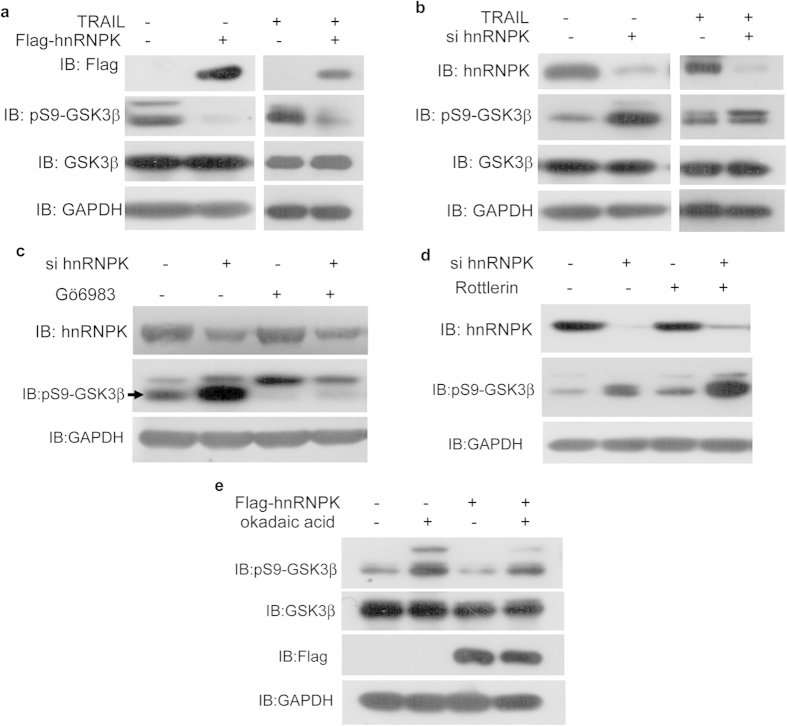
hnRNPK inhibited GSK3β Ser9 phosphorylation by PKC. H1299 cells respectively transfected with Flag-hnRNPK (**a**) or hnRNPK siRNA (**b**), and treated with or without TRAIL (20 ng/ml, 8 hours) were harvested for Western blot analysis with the indicated antibodies. (**c**) PKC inhibitor Gö6983 cancelled the regulatory effect of hnRNPK on GSK3β Ser9 phosphorylation. H1299 cells transfected with hnRNPK siRNA or/and treated with Gö6983 (1 μM) were analyzed by Western blotting with the indicated antibodies. (**d**) PKC inhibitor Rottlerin had no obvious effect on the regulation of GSK3β Ser9 phosphorylation by hnRNPK. H1299 cells transfected with hnRNPK siRNA or/and treated with Rottlerin (6 μM) were analyzed by Western blotting with the indicated antibodies. (**e**) PP1 inhibitor Okadaic acid did not affect the regulation of GSK3β Ser9 phosphorylation by hnRNPK. H1299 cells transfected with Flag-hnRNPK or/and treated with okadaic acid (100 nM) were analyzed by Western blotting with the indicated antibodies.

In our preliminary studies, we have observed that the overexpression of Flag-hnRNPK dramatically reduced the Ser9 phosphorylation of GSK3β in several kinds of cells, including H1299 cells (data not shown). This suggests that hnRNPK might inhibit the Ser9 phosphorylation of GSK3β and thereby regulate the TRAIL-induced apoptosis in H1299 cells. To confirm this hypothesis, we have either overexpressed Flag-hnRNPK or knocked down hnRNPK by siRNA in H1299 cells with or without TRAIL treatment, and examined the levels of total GSK3β and Ser9 phosphorylated GSK3β. As shown in Fig. 4a,b, the overexpression and depletion of hnRNPK respectively resulted in sharp inhibition and stimulation of GSK3β Ser9 phosphorylation, regardless the cells were treated or not with TRAIL, while the level of total GSK3β remained unaffected. This result clearly indicates that hnRNPK is capable of down-regulating the Ser9 phosphorylation of GSK3β.

Various serine/threonine kinases have been shown to be able to phosphorylate GSK3β on its serine 9 residue, including PKA, PKB/Akt, PKC, p70^S6K^, p90^RSK^/MAPKAP kinase-1^44^,^45^,^46^. Notably, PKC kinase has been reported to phosphorylate GSK3^33^,^47^,^48^,^49^ and positively regulate celecoxib-induced NSCLC cell apoptosis involving the activation of the death receptor pathway^33^. Therefore, we investigated if hnRNPK could inhibit the Ser9 phosphorylation of GSK3β by PKC in H1299 cells.

Firstly, we confirmed that the Ser9 phosphorylation of GSK3β in H1299 cells was mainly the effect of PKC kinase activity, since Gö6983, an inhibitor against the classic PKC α-ξ subtypes, was capable to inhibit the quasi totality of Ser9 phosphorylation of GSK3β in these cells (Fig. 4c). And more interestingly, with Gö6983 treatment, the knocking down of hnRNPK by siRNA had no longer obvious effect on the Ser9 phosphorylation of GSK3β. As contrast, when using rottlerin that is an inhibitor against the non-classic PKC δ subtype, hnRNPK siRNA still significantly stimulated the Ser9 phosphorylation of GSK3β (Fig. 4d). These results suggest that hnRNPK inhibits the Ser9 phosphorylation of GSK3β by PKC α-ξ but not PKC δ in H1299 cells. However, since the Ser9 phosphorylation state of GSK3β was regulated both by its phosphorylation by PKC and its dephosphorylation by PP1^50^, it remained to verify if hnRNPK might also stimulate the dephosphorylation of GSK3β by PP1. To address this issue, we have treated H1299 cells with 100 nM okadaic acid which inhibited the dephosphorylation of GSK3β by PP1, and examined the effect of hnRNPK expression on the phosphorylation state of GSK3β. Our result showed that the treatment by okadaic acid again did not alter the inhibitory effect of hnRNPK overexpression, excluding the possible regulation of GSK3β dephosphorylation by hnRNPK (Fig. 4e).

Taken together, these results suggest that hnRNPK is capable to inhibit the Ser9 phosphorylation of GSK3β by PKC, probably via steric hindrance caused by its interaction with GSK3β.

### hnRNPK inhibits GSK3β Ser9 phosphorylation, leading to the stabilization of c-FLIP protein and inactivation of Caspase 8 during the TRAIL-induced apoptosis of H1299 cells

**Figure 5:**
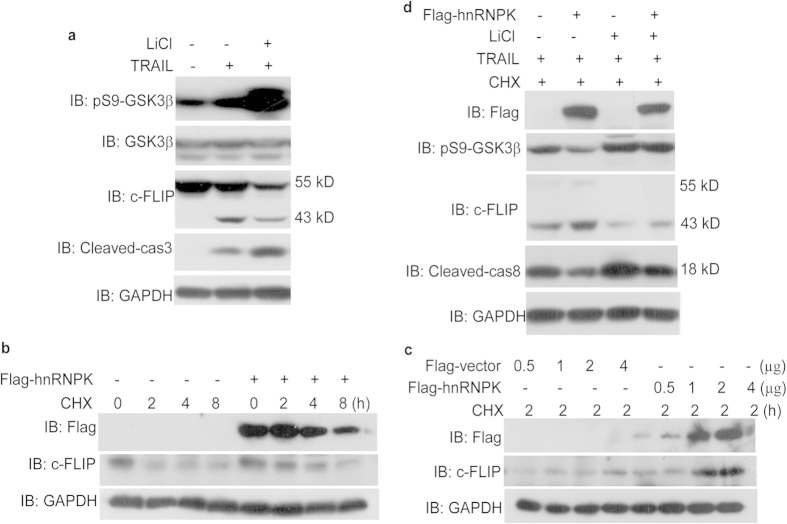
hnRNPK stabilized of c-FLIP protein through inhibition of GSK3β Ser9 phosphorylation during the TRAIL-induced apoptosis. (**a**) GSK3β upregulated the protein level of c-FLIP in H1299 cells treated with TRAIL. H1299 cells treated with LiCl (20 mM, 8 hours) and/or TRAIL (20 ng/ml, 8 hours) were harvested for Western blot analysis with the indicated antibodies. (**b**) Effect of hnRNPK overexpression on c-FLIP protein stability by Cycloheximide (CHX) chase experiments. H1299 cells transfected with either 2 μg Flag-hnRNPK or Flag-vector plasmids were treated with 10 μg/ml CHX for the indicated durations, then subjected to Western blot analysis with the indicated antibodies. (**c**) Effect of hnRNPK overexpression on c-FLIP protein stability by dose-response experiment. H1299 cells were transfected with Flag-hnRNPK or Flag-vector plasmids at the indicated doses and stimulated with CHX (10 μg/ml, 2 h), then subjected to Western blot analysis with the indicated antibodies. (**d**) Effect of hnRNPK on c-FLIP protein stability was dependent on the Ser9 phosphorylation state of GSK3β. H1299 cells transfected with Flag-hnRNPK or Flag-vector plasmids were stimulated with CHX (10 μg/ml, 2 h), TRAIL (20 ng/ml, 8 hours), or LiCl (20 mM, 8 hours) as indicated, then subjected to Western blot analysis with the indicated antibodies.

Based on the regulation of GSK3β Ser9 phosphorylation by hnRNPK as described hereinabove, and the stabilization of c-FLIP by GSK3β reported by others^33^, we searched to know if there was any link between these two events which might be part of the mechanisms involving these three proteins in the resistance of the TRAIL-induced apoptosis. We have firstly confirmed the regulatory role of GSK3β on the protein level of c-FLIP in H1299 cells treated with TRAIL. As shown in Fig. 5a, LiCl treatment of cells resulted in the increased level of GSK3β Ser9 phosphorylation and the down-regulation of both p55 and p43 c-FLIP protein levels, and as a consequence, the augmentation of the cleaved Caspase 3 amount. Next, we examined the effect of hnRNPK overexpression on c-FLIP protein stability in H1299 cells by performing cycloheximide chase experiments. H1299 cells transfected by either 2 μg Flag-hnRNPK or empty Flag plasmids were treated with 10 μg/ml cycloheximide (CHX). After various durations of treatment, cells were harvested and subjected to Western blotting analysis to assess the protein level of c-FLIP. Our results in Fig. 5b shows that in cells transfected with the empty Flag vector, most of the c-FLIP protein is degraded as rapidly as 2 hours after the treatment with CHX, while the overexpression of Flag-hnRNPK significantly slows down the c-FLIP degradation (Fig. 5b). The stabilizing effect of hnRNPK on c-FLIP protein was then further confirmed by a dose-response experiment. Cells transfected by increasing amount of either Flag-hnRNPK or empty Flag plasmids and treated with 10 μg/ml CHX during 2 hours were harvested. The protein level of c-FLIP was then assessed by Western blotting as described. As shown in Fig. 5c, the increasing concentration of overexpressed Flag-hnRNPK indeed resulted in the augmentation of c-FLIP in the cells as compared with the empty vector-transfected cells. And, as expected, the effect of hnRNPK was dependent on the Ser9 phosphorylation state of GSK3β, since LiCl treatment of the cells cancelled this effect. Moreover, c-FLIP expression levels are inversely correlated with those of the cleaved caspase 8, further confirming the functional effectiveness of this regulatory pathway (Fig. 5d). These results suggest that, in addition to the possible modulation of c-FLIP expression by hnRNPK at the transcriptional level as described in other report^41^, hnRNPK might also stabilize c-FLIP protein through regulation of GSK3β Ser9 phosphorylation.

### hnRNPK expression negatively correlates with Ser9 phosphorylated GSK3β in clinical lung adenocarcinoma tissues

**Figure 6:**
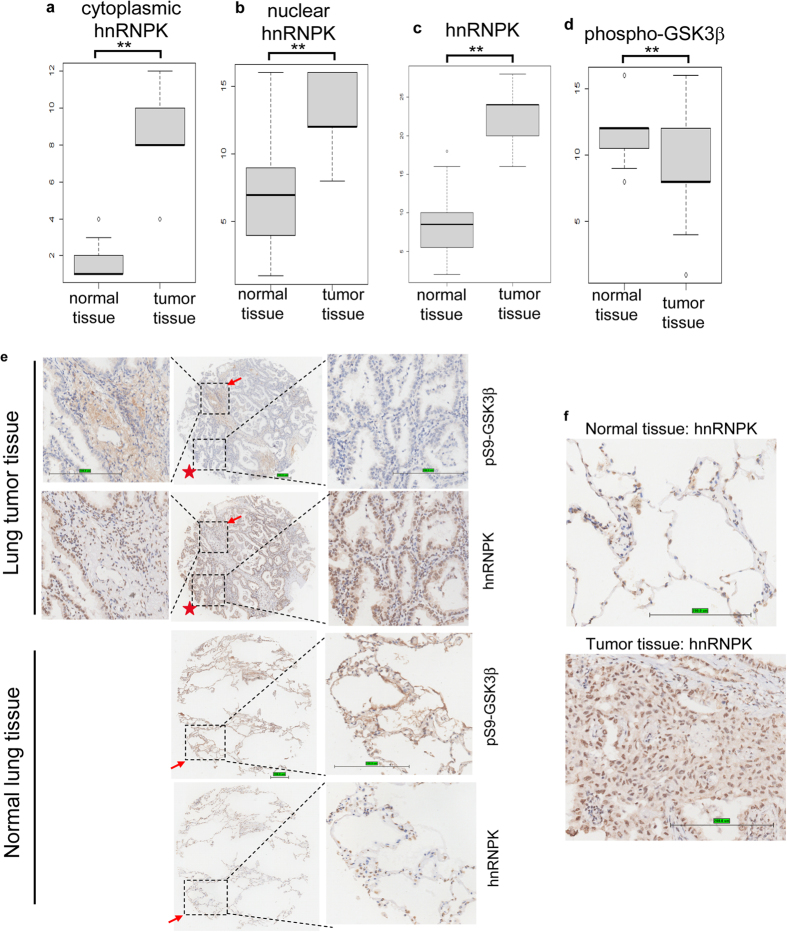
hnRNPK expression negatively correlates with Ser9 phosphorylated GSK3β in tissue microarrays (TMAs). TMAs of lung adenocarcinoma (n = 52) were immunohistochemically scored and statistically analyzed for the cytoplasmic hnRNPK (**a**), nuclear hnRNPK (**b**), total hnRNPK (**c**), and total phospho-GSK3β (**d**). **p < 0.001. (**e**) Immunohistochemical analysis of hnRNPK and phospho-GSK3β in representative normal lung tissue and lung tumor tissue on TMAs. The selected sections on the left and right panels were shown at 200× magnification. The panels in the middle were shown at 50× magnification. Brown color represents positive immune reaction. Scale bar: 200 μm. (**f**) Immunohistochemical analysis of hnRNPK in representative normal lung tissue and lung tumor tissue on TMAs. The selected portions were shown at 200× magnification. Scale bar: 200 μm.

In order to provide the clinical evidence of the regulation of GSK3β Ser9 phosphorylation by hnRNPK in lung cancer, we have performed the immunohistochemical essays using two commercialized tissue microarrays. 52 paired lung adenocarcinoma and adjacent normal samples were stained with either anti-hnRNPK antibody or anti-Ser9 phosphorylated GSK3β antibody, evaluated and scored as described in Materials and methods section. The results showed that hnRNPK was highly overexpressed in both the nucleus and cytoplasm of lung adenocarcinoma cells as compared with normal tissues (Fig. 6a–c), whereas the phospho-GSK3β was mainly located in the cytoplasm which was significantly downregulated in the tumor tissues (Fig. 6d). Furthermore, negative correlation between the level of hnRNPK and the Ser9 phosphorylated GSK3β could be established in both lung cancer and normal tissues (Fig. 6e). Strikingly, in the same histological section of lung cancer, we were able to simultaneously observe the low hnRNPK expression accompanied with the high level of phospho-GSK3β in a region (the arrows, Fig. 6e), and the opposite pattern in a neighbouring zone (the stars, Fig. 6e). Statistical analysis demonstrated that the expression levels of hnRNPK and phospho-GSK3β were inversely correlated in lung tumor tissues (p < 0.05, two-tailed). Finally, we found that in 52 (100%) carcinoma tissues examined, hnRNPK could be found in both the cytoplasm and nucleus whereas it was exclusively nuclear in normal tissues (Fig. 6f). This is consistent with another study performed by Pino and colleagues showing the cytoplasmic staining of hnRNPK in half of the 32 lung carcinoma samples examined^51^. This result strongly suggests the importance of the cytoplasmic hnRNPK in the genesis of lung adenocarcinoma, and its role in the regulation of the GSK3β Ser9-phosphorylation.

## Discussion

Owing to its capacity to specifically induce the apoptosis in tumor cells but not in normal cells, TRAIL is predicted to be one of the most effective anticancer therapeutic agents in the future^7^,^8^,^52^. However, resistance to TRAIL has turned out to be a general phenomenon and become a serious obstacle for the prospects of TRAIL therapy. The comprehensive understanding of the molecular mechanisms of TRAIL’s action thus reveals to be important and necessary for the development of combinatorial treatments involving TRAIL and others synergizing or sensitizing agents. Previous studies have established that the effects of TRAIL pass through the membrane death receptors 4 and 5 (DR4 and DR5) with their intracellular death domains, which activates dual opposing pathways leading respectively to the apoptosis but also the survival of the cell^52^. These two so called “apoptotic pathway” and “resistance pathway” are thought to be respectively responsible for the apoptotic effect of TRAIL and resistance to TRAIL in the cells. c-FLIP is regarded as the master anti-apoptotic regulator and resistance factor in TRAIL-induced apoptosis due to its effect of shifting the apoptotic pathway to the resistance pathway by facilitating the formation of the so called “secondary complex”. The observation that chemotherapeutic agents like celecoxib, camptothecin, and cisplatin could down regulate c-FLIP expression and sensitize the resistant cells to TRAIL treatment further demonstrated that c-FLIP plays a key role in the resistance to TRAIL-induced apoptosis and represents a potential target candidate for the combinatorial chemotherapeutic interventions with TRAIL^33^,^53^. c-FLIP has been shown to be a short-lived protein that is degraded via the proteasome pathway^17^,^23^,^54^. Therefore, the regulation of its stability might represent an interesting way to overcome the resistance to TRAIL. Our present study describes a novel mechanism of action of hnRNPK to antagonize TRAIL-induced apoptosis. hnRNPK is a DNA/RNA binding protein whose function has been so far associated with its nuclear localization and/or its regulation of the RNA expression and metabolism. We show here for the first time that the cytoplasmic hnRNPK interacts with GSK3β, and regulates its function to stabilize c-FLIP protein. Our finding is corroborated by our immunohistochemical analysis with tissue microarray demonstrating the negative correlation between hnRNPK expression level and that of the Ser9-phsphorylated GSK3β in lung adenocarcinoma tissues. Interestingly, hnRNPK has been previously reported to transcriptionally activate c-FLIP and antagonize TRAIL-induced apoptosis in nasopharyngeal carcinoma cells^41^. Taken together, these studies suggest that hnRNPK may up-regulate the c-FLIP protein level in cancer cells through two different mechanisms and at two different subcellular locations to antagonize the apoptotic effect of TRAIL, which would make this protein an interesting target for the combinatorial therapy with TRAIL.

GSK3β is a ubiquitous serine/threonine kinase with multiple functions involved in a wide range of cellular functions, including differentiation, growth, apoptosis, cell cycle, embryonic development, and insulin response^55^,^56^,^57^. The phosphorylation on the serine 9 residue of GSK3β is critical for its multiple functions. Our present study has permitted to identify hnRNPK protein as an efficient activator of GSK3β through inhibiting its Ser9 phosphorylation, one of the most active and versatile family of kinases responding to a variety of extracellular signals to regulate multiple signaling pathways in many types of cells^58^. Thus, in addition to its classic role of a DNA/RNA binding protein involved in the metabolism and expression of RNA, the discovery of its role as a regulator of GSK3β playing key roles in many signaling pathways and cellular processes turns out to be very exciting. The investigation of the possible regulation of GSK3β activity by hnRNPK in various physiological and pathological contexts reveals to be, in this regard, very interesting and important.

How could hnRNPK inhibit the phosphorylation state of GSK3β? Two possibilities could be taken into consideration. hnRNPK might either inhibit the phosphorylating activity of PKC, or facilitate the action of PP1 which dephosphorylates Ser9 phosphorylated GSK3β. The results of our experiments using the inhibitors respectively against PKC or PP1 have shown that hnRNPK acts more likely through the regulation of PKC rather than that of PP1 (Fig. 4c,e). The most probable scenario should be the stoichiometric hindrance caused by the hnRNPK- GSK3β interaction which renders inaccessible the Ser9 residue to PKC. We have attempted to verify this hypothesis by trying to assess the effect of hnRNPK overexpression on PKC-GSK3β interaction using co-immunoprecipitation assays. However, it turned out to be impossible to detect PKC-GSK3β interaction by this method (data not shown). Consistently, whilst the Ser9 phosphorylation of GSK3β by PKC has been well documented in the literature^33^,^47^,^48^,^49^, no report concerning the physical interaction between PKC and GSK3β could be found. This might probably due to the extremely dynamic interaction between these two proteins, as often observed between the kinases and their substrates. The verification of such hypothesis with more elaborated methodology would be of interest, and will be part of our future study.

## Materials and Methods

### Cell culture and transfection

Human non-small cell lung adenocarcinoma cell line H1299 (Cell Resource Center, Institute of life science Chinese Academy of Sciences, Shanghai, China) were cultured in Dulbecco’s modified Eagle medium (DMEM, Gibco BRL, Grand island, NY) supplemented with 10% fetal bovine serum (PAA Laboratories, Linz, Austria) at 37 °C in a humidified atmosphere containing 5% CO_2_. H1299 cells at a 80–90% confluence were transfected with Lipofectamin 2000 (Invitrogen, Carlsbad, CA).

### Expression and purification of GST fusion proteins

GST, GST-GSK3β, GST-GSK3β-S9A and GST-hnRNPK fusion proteins were produced and purified as described previously^34^. The protein concentrations were estimated by a BCA assay (Beyotime Biotechnology, China).

### Co-immunoprecipitation assay

H1299 cells transfected with Flag-hnRNPK or Flag-vector and treated with or without TRAIL (20 ng/ml, 8 hours) were lysed in lysis buffer (50 mM Tris at pH 7.5, 150 mM NaCl, 1% NP-40, 0.5% deoxycholate, 0.1% SDS, 5 mM NaF, 2 mM Na_3_VO_4_, 1 mM PMSF, 1% protease inhibitor cocktail (Roche)) for 30 min on ice. The co-immunoprecipitation assay were carried out with the protocols described previously^59^ using the GSK3β antibody (27C10, Cell Signaling Technology). The immune complexes were analyzed by Western blotting using antibodies against GSK3β and Flag (A2220, Sigma).

### Small interfering RNA assays

The sense strand sequences of siRNA used in this study are as follows: GSK3β siRNA: 5′-AAGUAAUCCACCUCUGGCUACTT-3′, hnRNPK siRNA1: 5′-UAUUAAGGCUCUCCGUACATT-3′, hnRNPK siRNA2: 5′-CCUUAΜGAUCCCAACUUUUTT-3′ and control siRNA (NC): 5′-UUCUCCGAACGΜGUCACGUTT-3′. These siRNAs were synthetized in GenePharma (Shanghai, China) and transfected into H1299 cells at 100 nM using Lipofectamin 2000 (Invitrogen). hnRNPK siRNA1 and hnRNPK siRNA2 were used in mixture. The interfered cells were harvested 48 hours after transfection.

### GST pull-down assays

The GST pull-down assays were performed as described previously^34^,^60^,^61^. Briefly, 1.5 mg whole cell lysate protein was incubated with 60 μg GST-hnRNPK protein overnight at 4 °C on a rocker. Cellular proteins associated with GST-tagged fusion proteins were separated by SDS-PAGE, and analyzed by standard Western blotting using specific rabbit monoclonal GSK3β antibody (27C10, Cell Signaling Technology, MA), phospho-GSK3β (Ser9) antibody (9323, Cell Signaling Technology, MA). The amounts of GST-tagged fusion proteins were estimated by CBB (Coomassie Brilliant Blue) staining of SDS-PAGE gels.

### MTT assays for cellular viability

Cellular viability was evaluated with the MTT (3-(4,5-dimethylthiazol-2-yl)-2,5-diphenyltetrazolium bromide, Beyotime Biotechnology, China) assay. H1299 cells (4000 cells per well) were seed into 96-well plate (TCP011096, JET, Guangzhou, China) and incubated for 16 h. Then, the cells were treated with TRAIL or/and LiCl (Sigma, 20 mM, 8 hours) as indicated. After treatments, the cells were incubated with MTT solution (final concentration 20 μg/ml) for 4 h at 37 °C and the produced formazan crystals were dissolved in 150 μl DMSO per well. Cellular viability was determined by measuring the absorbance of formazan crystals at 570 nm using a spectrophotometric microplate reader (ELX800, BioTek Instrument, USA). Three independent experiments were performed. The cellular viability of untreated controls was set as 100%.

### Flow cytometry

H1299 cells after transfection of Flag-hnRNPK or knockdown of GSK3β were treated with TRAIL (20 ng/ml, 8 hours) as indicated, then the cells were collected and stained with Alexa Fluor 488 Annexin-V/PI (HH-V13241, Invitrogen) following the manufacturer’s protocol. Cells were subsequently sorted by a FACS Calibur flow cytometer (BD Biosciences) and analyzed by Flowjo software. Only living cells profiled using forward scatter (FSC) and side scatter (SSC) were counted. At least 10,000 cells within the gated region were counted and analyzed.

### Detection of protein expression by Western blotting

Cells were transfected with Flag-GSK3β, Flag-hnRNPK, siRNA target against GSK3β or hnRNPK, or combined with treatments of TRAIL (Sino Biological Inc. Beijing, China), LiCl (Sigma), Gö6983 (1 μM, Beyotime, China), Rottlerin (6 μM, Millipore, USA), or CHX (10 μg/ml, Amresco, USA) when it is indicated. Cells were then harvested and lysed with lysis buffer (20 mM Tris (pH7.5), 150 mM NaCl, 1% Triton X-100, sodium pyrophosphate, β-glycerophosphate, EDTA, Na_3_VO_4_, leupeptin, and 1% protease inhibitor cocktail (Roche)). Whole cell lysates were separated by 10% or 12% SDS/PAGE.

Blots were probed with the specific antibodies against Flag (A2220, Sigma), GSK3β (27C10, Cell Signaling Technology), phospho-GSK3β (Ser9) (Cell Signaling Technology), hnRNPK (sc-28380, SANTA CRUZ), GAPDH (ZS-25778, ZSGB-BIO, China), β-catenin (Beyotime, China), Cleaved Caspase 3 (9664, Cell Signaling Technology), Cleaved Caspase 8 (9496, Cell Signaling Technology), c-FLIP (7F10, Alexis, Enzo life science, Switzerland). Horseradish peroxidase-conjugated secondary antibodies (ProteinTech Group) and enhanced chemiluminescence (ECL kit, Beyotime, China) were used to detect the expression of proteins.

### Confocal microscopy assay

H1299 lung adenocarcinoma cells transfected with or without Flag-hnRNPK were treated with TRAIL (20 ng/ml). Immunofluorescence was performed as described previously^34^,^60^. The primary antibodies (1:100 dilution) of hnRNPK, Flag and GSK3β, and the Alexa Fluor 488 and Alexa Fluor 594 conjugated secondary antibodies (ZSGB-BIO, China) were used for observation. DAPI staining was used to determine the morphology of cell nuclei.

The imaging experiments were carried out on laser scanning confocal microscopes (LSM700, Zeiss, Jena, Germany) equipped with a Zeiss Plan-Neofluar 40×/1.3 NA Oil Dic objective as described previously^34^,^60^.

### Tissue microarrays and Statistics analysis

The expression of hnRNPK and the Ser9 phosphorylation of GSK3β was analyzed using two consecutively numbered commercial tissue microarrays (TMAs) of lung adenocarcinoma (catalog no. OD-CT-RsLug04-003, Shanghai Outdo Biotech, Shanghai, China), consisting of 53 paired lung adenocarcinoma and adjacent normal samples, 2 unpaired lung adenocarcinoma tissues. The TMAs came from 30 male and 25 female patients, and were mainly grade II in the pathological grading. During the experimental process, one sample of lung adenocarcinoma tissue was destroyed, the final 52 paired samples of adenocarcinoma and normal tissues were analyzed.

The immunohistochemical staining of TMAs were performed in Shanghai Outdo Biotech. Briefly, tissue sections (4 μm) were incubated in a 63 °C oven for one hour. After deparaffnization and rehydration, antigen retrieval was carried out by boiling sections in citrate buffer (10 mM, pH 6.0) for 5 min. Then, endogenous peroxidase activity was blocked with 3% H_2_O_2_ for 15 min at room temperature. The slides were blocked with 3% BSA in PBS for 30 min and subsequently cultured with primary antibodies against phospho-GSK3β (Ser9) (1:100 diluion) and hnRNPK (1:500 diluion), respectively. The immune complex was detected using a Dako EnVision™ Detection system (Dako Japan Ltd.). Nuclei were counterstained with hematoxylin. Images of TMAs were captured by Aperio Scanscope XT (Leica, Germany).

The results were evaluated independently by 2 pathologists according to both the intensity of protein staining and the percentage of positive cells. The intensity of protein staining was scored as 1 (negative), 2 (weak), 3 (moderate) or 4 (strong). Evaluation of the percentage of positive cells was scored as: 1 (0%), 2 (≤30%), 3 (31–60%), and 4 (>60%). The total score of each case was consisting of the score of the intensity of protein staining and the score of percentage of positive cells. The paired Student’s t-test was employed to analyze the significance of phospho-GSK3β or hnRNPK with tumor or pericancerous normal tissue. Correlation between the expression of hnRNPK and phospho-GSK3β (Ser9) were estimated using the Spearman’s rank correlation analysis.

## Additional Information

**How to cite this article**: Gao, X. *et al.* hnRNPK inhibits GSK3β Ser9 phosphorylation, thereby stabilizing c-FLIP and contributes to TRAIL resistance in H1299 lung adenocarcinoma cells. *Sci. Rep.* **6**, 22999; doi: 10.1038/srep22999 (2016).

## Supplementary Material

Supplementary Information

## References

[b1] Jemal A. *et al.* Cancer statistics, 2006. CA. Cancer J. Clin. 56, 106–130 (2006). 16514137 10.3322/canjclin.56.2.106

[b2] Erridge S. C., Moller H., Price A. & Brewster D. International comparisons of survival from lung cancer: pitfalls and warnings. Nat. Clin. Pract. Oncol. 4, 570–577 (2007). 17898807 10.1038/ncponc0932

[b3] Wagner K. W. *et al.* Activation and suppression of the TRAIL death-receptor pathway in chemotherapy sensitive and resistant follicular lymphoma cells. Cancer Biol. Ther. 2, 534–540 (2003). 14614322 10.4161/cbt.2.5.453

[b4] Voortman J., Resende T. P., Abou El Hassan M. A., Giaccone G. & Kruyt F. A. TRAIL therapy in non-small cell lung cancer cells: sensitization to death receptor-mediated apoptosis by proteasome inhibitor bortezomib. Mol. Cancer Ther. 6, 2103–2112 (2007). 17620439 10.1158/1535-7163.MCT-07-0167

[b5] Amm H. M., Oliver P. G., Lee C. H., Li Y. & Buchsbaum D. J. Combined modality therapy with TRAIL or agonistic death receptor antibodies. Cancer Biol. Ther. 11, 431–449 (2011). 21263219 10.4161/cbt.11.5.14671 PMC3087899

[b6] Goncharenko-Khaider N., Matte I., Lane D., Rancourt C. & Piché A. Ovarian cancer ascites increase Mcl-1 expression in tumor cells through ERK1/2-Elk-1 signaling to attenuate TRAIL-induced apoptosis. Mol. Cancer 11, 84 (2012). 23158473 10.1186/1476-4598-11-84 PMC3526430

[b7] Zhuang H. *et al.* Suppression of HSP70 expression sensitizes NSCLC cell lines to TRAIL-induced apoptosis by upregulating DR4 and DR5 and downregulating c-FLIP-L expressions. J. Mol. Med (Berl). 91, 219–235 (2013). 22948392 10.1007/s00109-012-0947-3

[b8] Walczak H. *et al.* Tumoricidal activity of tumor necrosis factor-related apoptosis-inducing ligand *in vivo*. Nat. Med. 5, 157–163 (1999). 9930862 10.1038/5517

[b9] Johnstone R. W., Frew A. J. & Smyth M. J. The TRAIL apoptotic pathway in cancer onset, progression and therapy. Nat. Rev. Cancer 8, 782–798 (2008). 18813321 10.1038/nrc2465

[b10] Todaro M. *et al.* Apoptosis resistance in epithelial tumors is mediated by tumor-cell-derived interleukin-4. Cell Death Differ. 15, 762–772 (2008). 18202702 10.1038/sj.cdd.4402305

[b11] Felber M., Sonnemann J. & Beck J. F. Inhibition of novel protein kinase C-epsilon augments TRAIL-induced cell death in A549 lung cancer cells. Pathol. Oncol. Res. 13, 295–301 (2007). 18158564 10.1007/BF02940308

[b12] Kischkel F. C. *et al.* Apo2L/TRAIL-dependent recruitment of endogenous FADD and caspase-8 to death receptors 4 and 5. Immunity 12, 611–620 (2000). 10894161 10.1016/s1074-7613(00)80212-5

[b13] Wiley S. R. *et al.* Identification and characterization of a new member of the TNF family that induces apoptosis. Immunity 3, 673–682 (1995). 8777713 10.1016/1074-7613(95)90057-8

[b14] Peter M. E. & Krammer P. H. The CD95(APO-1/Fas) DISC and beyond. Cell Death Differ. 10, 26–35 (2003). 12655293 10.1038/sj.cdd.4401186

[b15] Kaufmann S. H. & Hengartner M. O. Programmed cell death: alive and well in the new millennium. Trends Cell Biol. 11, 526–534 (2001). 11719060 10.1016/s0962-8924(01)02173-0

[b16] Wajant H. Targeting the FLICE Inhibitory Protein (FLIP) in cancer therapy. Mol. Interv. 3, 124–127 (2003). 14993418 10.1124/mi.3.3.124

[b17] Safa A. R. & Pollok K. E. Targeting the anti-apoptotic protein c-FLIP for cancer therapy. Cancers (Basel) 3, 1639–1671 (2011). 22348197 10.3390/cancers3021639 PMC3281420

[b18] Safa A. R. c-FLIP, a master anti-apoptotic regulator. Exp. Oncol. 34, 176–184 (2012). 23070002 PMC4817998

[b19] Li B. *et al.* The novel Akt inhibitor API-1 induces c-FLIP degradation and synergizes with TRAIL to augment apoptosis independent of Akt inhibition. Cancer Prev. Res (Phila). 5, 612–620 (2012). 22345097 10.1158/1940-6207.CAPR-11-0548 PMC3324640

[b20] Ullenhag G. J. *et al.* Overexpression of FLIPL is an independent marker of poor prognosis in colorectal cancer patients. Clin. Cancer Res. 13, 5070–5075 (2007). 17785559 10.1158/1078-0432.CCR-06-2547

[b21] Korkolopoulou P. *et al.* c-FLIP expression in bladder urothelial carcinomas: its role in resistance to Fas-mediated apoptosis and clinicopathologic correlations. Urology 63, 1198–1204 (2004). 15183989 10.1016/j.urology.2004.01.007

[b22] Yang J. K. FLIP as an anti-cancer therapeutic target. Yonsei. Med. J. 49, 19–27 (2008). 18306465 10.3349/ymj.2008.49.1.19 PMC2615266

[b23] Bagnoli M., Canevari S. & Mezzanzanica D. Cellular FLICE-inhibitory protein (c-FLIP) signalling: a key regulator of receptor-mediated apoptosis in physiologic context and in cancer. Int. J. Biochem. Cell Biol. 42, 210–213 (2010). 19932761 10.1016/j.biocel.2009.11.015

[b24] Ueffing N. *et al.* A single nucleotide polymorphism determines protein isoform production of the human c-FLIP protein. Blood 114, 572–579 (2009). 19439735 10.1182/blood-2009-02-204230

[b25] Kump E., Ji J., Wernli M., Häusermann P. & Erb P. Gli2 upregulates cFlip and renders basal cell carcinoma cells resistant to death ligand-mediated apoptosis. Oncogene 27, 3856–3864 (2008). 18264131 10.1038/onc.2008.5

[b26] Fang L. W., Tai T. S., Yu W. N., Liao F. & Lai M. Z. Phosphatidylinositide 3-kinase priming couples c-FLIP to T cell activation. J. Biol. Chem. 279, 13–18 (2004). 14578361 10.1074/jbc.M303860200

[b27] Dohrman A. *et al.* Cellular FLIP (long form) regulates CD8+ T cell activation through caspase-8-dependent NF-kappa B activation. J. Immunol. 174, 5270–5278 (2005). 15843523 10.4049/jimmunol.174.9.5270

[b28] Beurel E. & Jope R. S. The paradoxical pro- and anti-apoptotic actions of GSK3 in the intrinsic and extrinsic apoptosis signaling pathways. Prog. Neurobiol. 79, 173–189 (2006). 16935409 10.1016/j.pneurobio.2006.07.006 PMC1618798

[b29] Kotliarova S. *et al.* Glycogen synthase kinase-3 inhibition induces glioma cell death through c-MYC, nuclear factor-kappaB, and glucose regulation. Cancer Res. 68, 6643–6651 (2008). 18701488 10.1158/0008-5472.CAN-08-0850 PMC2585745

[b30] Beurel E. *et al.* Glycogen synthase kinase-3 inhibitors augment TRAIL-induced apoptotic death in human hepatoma cells. Biochem. Pharmacol. 77, 54–65 (2009). 18938143 10.1016/j.bcp.2008.09.026

[b31] Liao X., Zhang L., Thrasher J. B., Du J. & Li B. Glycogen synthase kinase-3beta suppression eliminates tumor necrosis factor-related apoptosis-inducing ligand resistance in prostate cancer. Mol. Cancer Ther. 2, 1215–1222 (2003). 14617795

[b32] Song L., Zhou T. & Jope R. S. Lithium facilitates apoptotic signaling induced by activation of the Fas death domain-containing receptor. BMC Neurosci. 5, 20 (2004). 15157283 10.1186/1471-2202-5-20 PMC420462

[b33] Chen S. *et al.* Celecoxib promotes c-FLIP degradation through Akt-independent inhibition of GSK3. Cancer Res. 71, 6270–6281 (2011). 21868755 10.1158/0008-5472.CAN-11-0838 PMC3185138

[b34] Gao X., Wang J. Y., Gao L. M., Yin X. F. & Liu L. Identification and analysis of glycogen synthase kinase 3 beta1 interactome. Cell Biol. Int. 37, 768–779 (2013). 23505128 10.1002/cbin.10095

[b35] Choi H. S. *et al.* Poly(C)-binding proteins as transcriptional regulators of gene expression. Biochem. Biophys. Res. Commun. 380, 431–436 (2009). 19284986 10.1016/j.bbrc.2009.01.136 PMC2657093

[b36] Lee P. T., Liao P. C., Chang W. C. & Tseng J. T. Epidermal growth factor increases the interaction between nucleolin and heterogeneous nuclear ribonucleoprotein K/poly(C) binding protein 1 complex to regulate the gastrin mRNA turnover. Mol. Biol. Cell 18, 5004–5013 (2007). 17928403 10.1091/mbc.E07-04-0384 PMC2096583

[b37] Barboro P. *et al.* Heterogeneous nuclear ribonucleoprotein K: altered pattern of expression associated with diagnosis and prognosis of prostate cancer. Br. J. Cancer 100, 1608–1616 (2009). 19401687 10.1038/sj.bjc.6605057 PMC2696760

[b38] Matta A. *et al.* Heterogeneous ribonucleoprotein K is a marker of oral leukoplakia and correlates with poor prognosis of squamous cell carcinoma. Int. J. Cancer 125, 1398–1406 (2009). 19548310 10.1002/ijc.24517

[b39] Chen L. C. *et al.* Heterogeneous ribonucleoprotein k and thymidine phosphorylase are independent prognostic and therapeutic markers for nasopharyngeal carcinoma. Clin. Cancer Res. 14, 3807–3813 (2008). 18559600 10.1158/1078-0432.CCR-08-0155

[b40] Carpenter B. *et al.* Heterogeneous nuclear ribonucleoprotein K is over expressed, aberrantly localised and is associated with poor prognosis in colorectal cancer. Br. J. Cancer 95, 921–927 (2006). 16953238 10.1038/sj.bjc.6603349 PMC2360539

[b41] Chen L. C. *et al.* The antiapoptotic protein, FLIP, is regulated by heterogeneous nuclear ribonucleoprotein K and correlates with poor overall survival of nasopharyngeal carcinoma patients. Cell Death Differ. 17, 1463–1473 (2010). 20224598 10.1038/cdd.2010.24

[b42] Jope R. S. Lithium and GSK-3: one inhibitor, two inhibitory actions, multiple outcomes. Trends Pharmacol. Sci. 24, 441–443 (2003). 12967765 10.1016/S0165-6147(03)00206-2

[b43] Rottmann S., Wang Y., Nasoff M., Deveraux Q. L. & Quon K. C. A TRAIL receptor-dependent synthetic lethal relationship between MYC activation and GSK3beta/FBW7 loss of function. Proc. Natl. Acad. Sci. USA 102, 15195–15200 (2005). 16210249 10.1073/pnas.0505114102 PMC1257707

[b44] Sutherland C. & Cohen P. The alpha-isoform of glycogen synthase kinase-3 from rabbit skeletal muscle is inactivated by p70 S6 kinase or MAP kinase-activated protein kinase-1 *in vitro*. FEBS Lett. 338, 37–42 (1994). 8307153 10.1016/0014-5793(94)80112-6

[b45] Cross D. A., Alessi D. R., Cohen P., Andjelkovich M. & Hemmings B. A. Inhibition of glycogen synthase kinase-3 by insulin mediated by protein kinase B. Nature 378, 785–789 (1995). 8524413 10.1038/378785a0

[b46] Eldar-Finkelman H., Seger R., Vandenheede J. R. & Krebs E. G. Inactivation of glycogen synthase kinase-3 by epidermal growth factor is mediated by mitogen-activated protein kinase/p90 ribosomal protein S6 kinase signaling pathway in NIH/3T3 cells. J. Biol. Chem. 270, 987–990 (1995). 7836418 10.1074/jbc.270.3.987

[b47] Wang Q., Zhou Y. & Evers B. M. Neurotensin phosphorylates GSK-3al-pha/beta through the activation of PKC in human colon cancer cells. Neoplasia 8, 781–787 (2006). 16984735 10.1593/neo.06259 PMC1584301

[b48] Shin S. Y., Yoon S. C., Kim Y. H., Kim Y. S. & Lee Y. H. Phosphorylation of glycogen synthase kinase-3beta at serine-9 by phospholipase Cgamma1 through protein kinase C in rat 3Y1 fibroblasts. Exp. Mol. Med. 34, 444–450 (2002). 12526086 10.1038/emm.2002.62

[b49] Fang X. *et al.* Convergence of multiple signaling cascades at glycogen synthase kinase 3: Edg receptor-mediated phosphorylation and inactivation by lysophosphatidic acid through a protein kinase C-dependent intracellular pathway. Mol. Cell. Biol. 22, 2099–2110 (2002). 11884598 10.1128/MCB.22.7.2099-2110.2002 PMC133668

[b50] Hernández F., Langa E., Cuadros R., Avila J. & Villanueva N. Regulation of GSK3 isoforms by phosphatases PP1 and PP2A. Mol. Cell. Biochem. 344, 211–215 (2010). 20652371 10.1007/s11010-010-0544-0

[b51] Pino I. *et al.* Altered patterns of expression of members of the heterogeneous nuclear ribonucleoprotein (hnRNP) family in lung cancer. Lung Cancer 41, 131–143 (2003). 12871776 10.1016/s0169-5002(03)00193-4

[b52] Refaat A., Abd-Rabou A. & Reda A. TRAIL combinations: The new ‘trail’ for cancer therapy (Review). Oncol. Lett. 7, 1327–1332 (2014). 24765133 10.3892/ol.2014.1922 PMC3997674

[b53] Wang P. *et al.* Inhibition of RIP and c-FLIP enhances TRAIL-induced apoptosis in pancreatic cancer cells. Cell. Signal. 19, 2237–2246 (2007). 17693058 10.1016/j.cellsig.2007.06.001

[b54] Safa A. R., Day T. W. & Wu C. H. Cellular FLICE-like in¬hibitory protein (c-FLIP): A novel target for cancer therapy. Curr. Cancer Drug Targets 8, 37–46 (2008). 18288942 10.2174/156800908783497087 PMC4524510

[b55] Sanchez J. F. *et al.* Glycogen synthase kinase 3beta-mediated apoptosis of primary cortical astrocytes involves inhibition of nuclear factor kappaB signaling. Mol. Cell. Biol. 23, 4649–4662 (2003). 12808104 10.1128/MCB.23.13.4649-4662.2003 PMC164840

[b56] Luo J. Glycogen synthase kinase 3beta (GSK3beta) in tumorigenesis and cancer chemotherapy. Cancer Lett. 273, 194–200 (2009). 18606491 10.1016/j.canlet.2008.05.045 PMC4978950

[b57] Jacobs K. M. *et al.* GSK-3β: A Bifunctional Role in Cell Death Pathways. Int. J. Cell Biol. 2012, 930710 (2012). 10.1155/2012/930710 PMC3364548 22675363

[b58] Kang J.-H. Protein Kinase C (PKC) Isozymes and Cancer. New J. Sci. 2014, 1–36 (2014).

[b59] Gao X., Wang J. Y., Gao L. M., Yin X. F. & Liu L. Identification and analysis of glycogen synthase kinase 3 beta1 interactome. Cell Biol Int 37, 768–779 (2013). 23505128 10.1002/cbin.10095

[b60] Gao X. *et al.* 14-3-3ζ reduces DNA damage by interacting with and stabilizing proliferating cell nuclear antigen. J. Cell. Biochem. 116, 158–169 (2015). 25169136 10.1002/jcb.24955

[b61] Gao X. *et al.* Ser9-phosphorylated GSK3β induced by 14-3-3ζ actively antagonizes cell apoptosis in a NF-κB dependent manner. Biochem. Cell Biol. 92, 349–356 (2014). 25138042 10.1139/bcb-2014-0065

