# Supplementary material for: hnRNPK inhibits GSK3β Ser9 phosphorylation, thereby stabilizing c-FLIP and contributes to TRAIL resistance in H1299 lung adenocarcinoma cells
Source: Sci Rep. 2016 Mar 14;6:22999. doi: 10.1038/srep22999 (PMC4789638; doi:10.1038/srep22999)

**hnRNPK inhibits GSK3β Ser9 phosphorylation, thereby stabilizing c-FLIP and contributes to TRAIL resistance in H1299 lung adenocarcinoma cells**

Xuejuan Gao^1,+^, Junxia Feng^2,+^, Yujiao He^1,+^, Fengmei Xu^1^, Xiaoqin Fan^3^, Wensi Huang^1^, Haiting Xiong^1^, Qiuyu Liu^4^, Wanting Liu^1^, Xiaohui Liu^1^, Xuesong Sun^1^, Qing-Yu He^1^, Qihao Zhang^5,*^, and Langxia Liu^1,*^

**Supplementary figure**

**Figure S1**

**Ser9 phophorylated and unphosphorylated GSK3β interact equally with hnRNPK.**

GST, GST-GSK3β, and GST-GSK3β-S9A fusion proteins were produced and purified as described previously^34^. GST-pull down assays were performed with 50 μg GST, GST-GSK3β or GST-GSK3β-S9A fusion proteins and 1 mg protein extract of H1299 cells similarly as described in the Materials and methods section of the manuscript.

GST

GST-3β

GST-3β-S9A

Input

WB：hnRNPK

Coomaissie


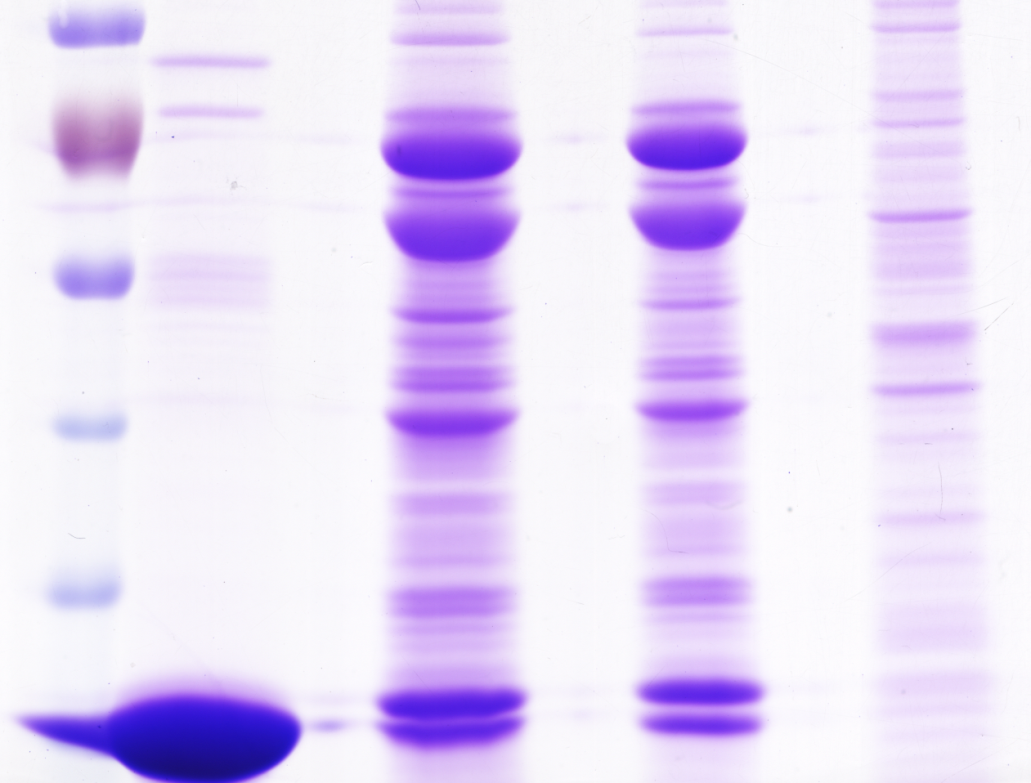

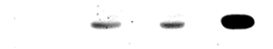

Supplement: Supplementary Information [file srep22999-s1.doc]
